# Sensitive Analysis of Idarubicin in Human Urine and Plasma by Liquid Chromatography with Fluorescence Detection: An Application in Drug Monitoring

**DOI:** 10.3390/molecules25245799

**Published:** 2020-12-09

**Authors:** Olga Maliszewska, Natalia Treder, IIona Olędzka, Piotr Kowalski, Natalia Miękus, Tomasz Bączek, Wojciech Rodzaj, Ewa Bień, Małgorzata Anna Krawczyk, Alina Plenis

**Affiliations:** 1 Department of Pharmaceutical Chemistry, Medical University of Gdańsk, Hallera 107, 80-416 Gdańsk, Poland; olgam@gumed.edu.pl (O.M.); natalia.treder@gumed.edu.pl (N.T.); ilona@gumed.edu.pl (I.O.); piotr.kowalski@gumed.edu.pl (P.K.); miekusn@gumed.edu.pl (N.M.); tbaczek@gumed.edu.pl (T.B.); 2 Department of Toxicology, Medical University of Gdańsk, Hallera 107, 80-416 Gdańsk, Poland; wojciech.rodzaj@gumed.edu.pl; 3 Department of Pediatrics, Hematology and Oncology, Medical University Gdańsk, Dębinki 7, 80-210 Gdańsk, Poland; ebien@gumed.edu.pl (E.B.); mkrawczyk@gumed.edu.pl (M.A.K.)

**Keywords:** anthracyclines, idarubicin, human and urine samples, liquid-liquid extraction (LLE), solid-phase extraction (SPE), solid-phase microextraction (SPME), liquid chromatography with fluorescence detection (LC-FL), drug monitoring

## Abstract

A new approach for the sensitive, robust and rapid determination of idarubicin (IDA) in human plasma and urine samples based on liquid chromatography with fluorescence detection (LC-FL) was developed. Satisfactory chromatographic separation of the analyte after solid-phase extraction (SPE) was performed on a Discovery HS C18 analytical column using a mixture of acetonitrile and 0.1% formic acid in water as the mobile phase in isocratic mode. IDA and daunorubicin hydrochloride used as an internal standard (I.S.) were monitored at the excitation and emission wavelengths of 487 and 547 nm, respectively. The method was validated according to the FDA and ICH guidelines. The linearity was confirmed in the range of 0.1–50 ng/mL and 0.25–200 ng/mL, while the limit of detection (LOD) was 0.05 and 0.125 ng/mL in plasma and urine samples, respectively. The developed LC-FL method was successfully applied for drug determinations in human plasma and urine after oral administration of IDA at a dose of 10 mg to a patient with highly advanced alveolar rhabdomyosarcoma (RMA). Moreover, the potential exposure to IDA present in both fluids for healthcare workers and the caregivers of patients has been evaluated. The present LC-FL method can be a useful tool in pharmacokinetic and clinical investigations, in the monitoring of chemotherapy containing IDA, as well as for sensitive and reliable IDA quantitation in biological fluids.

## 1. Introduction

Anthracyclines are a group of antibiotics with cytostatic activity. They are the most frequently used and the most effective anticancer drugs. This category includes idarubicin (IDA), a derivative of daunorubicin (DAU) (Supplementary Materials, Figure S1) belonging to the group of second-generation drugs [1,2]. This substance was approved by the United States Food Drug Administration (US FDA) in 1990 and is widely used as a hydrochloride [3]. The mechanism of action of IDA is similar to the other anthracyclines, but higher lipophilicity results in easier formation of the complex with topoisomerase II, which causes DNA strand breaks [4,5], IDA has properties that distinguish it from the most commonly used anthracycline antibiotics such as doxorubicin, epirubicin and DAU. In turn, because of greater lipophilicity, it is possible to use it not only intravenously, but also orally [6,7,8]. This substance is active against multiple myeloma, non-Hodgkin’s lymphoma and advanced breast cancer [2,5,6,9,10]. It is also indicated for use in acute myeloid leukemia and rhabdomyosarcoma (RMA) [1,11]. There are many different IDA dosing schedules [2,4,8,11,12]. One example is the scheme of oral (O) trofosfamide (T), IDA and etoposide (E) (O-TIDA/O-TE) maintenance therapy, during which IDA is administered at a dose of 5 mg/m^2^/day p.o., once a day, on days 1, 4, 7 and 10. The above scheme is used during RMA treatment [13]. Many chemotherapy regimens are based on the combination of several cytostatic drugs with different mechanisms of action. In turn, the use of cytostatics during treatment carries a significant risk of side effects. Despite the fact that IDA is less cardiotoxic than other anthracyclines like DAU and doxorubicin, drug monitoring should be conducted to prevent side effects as well as for the improvement of chemotherapy results [1,2,4,5]. Thus, it is necessary to develop a fast and reliable analytical method for drug determination in body fluids, which can be an indispensable element of such therapy. Of course, each developed method should be adjusted to the specific pharmacokinetic and clinical application. For example, when a high drug dose is administrated to a patient intravenously, the analytical method should offer a wide linearity range, while the limit of detection (LOD) and limit of quantification (LOQ) may be higher. In the case of oral drug administration at a low dose, significantly lower LOD and LOQ parameters are required, whereas a shorter range of linearity can be sufficient for a given application.

In the scientific literature, there are several reports which describe analytical methods based on liquid chromatography (LC) [6,7,9,14,15,16,17,18,19,20] (Table S1), electrophoretic techniques [21,22] and electrochemical sensor approaches [23,24,25] for IDA determination in various biological matrices. Among LC approaches, mainly reported between 1990 and 2012, IDA was quantified in human [2,6,7,8,15,16,18] and animal plasma samples [19,20]. The compound was also analyzed in human urine [7,14,18], saliva [16], serum [17], and other biological matrices [9]. Liquid-liquid extraction (LLE) was often used for IDA isolation from tested biological matrices [15,16,20] although protein precipitation was also applied [15]. Solid-phase extraction (SPE) was also commonly reported [6,7,9,14,17,18]. However, many sample preparation protocols described in the literature were tedious and time-consuming [7,15,16], the extraction efficiency for IDA was low [9,16] or the presented results were not absolute recovery results [6,7]. According to the literature data, IDA was monitored mainly by fluorescence (FL) detection with an excitation radiation wavelength in the 460–485 nm range and emission in the 540–580 nm range [9,15,16,18,19,20]. In three papers, excitation radiation with a wavelength of 230 [15], 250 nm [6] and 254 nm [7,15] was used. Another approach was laser-induced fluorescence (LIF) detection at 488 nm used in the capillary electrophoresis (CE) [22]. The common usage of FL detection for IDA determination in biological fluids is related to the fact that this molecule possesses natural fluorescence activity and no derivative step is required during sample preparation. Additionally, a significantly lower LOD and LOQ can be obtained compared to those with ultraviolet (UV), which are frequently insufficient for IDA quantification in biological samples. Moreover, LC methods coupled to mass spectrometry (MS) [14,17] were also applied. Among the developed methods, the widest linearity range obtained was 0.4–10,000 ng/mL [15]. The lowest LOD value was found to be 0.1 ng/mL in human plasma samples, but full validation report was not presented [6]. Unfortunately, previous reported methodologies can be difficult for use in routine clinical analysis because of their long analysis time (≥15 min) [14,15,17,18,19,21], required large sample volume, which can be difficult to obtain from pediatric patients [6,7,9,14,18], or too high LOQ parameters for specific clinical applications (≥0.5 ng/mL) [14,16,17,20,22]. Moreover, the usefulness of the reported methods based on biosensors was not confirmed in clinical practice [23,24,25]. Furthermore, to the best of our knowledge, there are no literature reports regarding the use of solid-phase microextraction (SPME), as well as SPE based on other than C18 cartridges, as potential techniques for isolating IDA from human body fluids. There is also a small number of publications which have reported measuring IDA levels in plasma and urine from pediatric oncological patients after an oral intake of IDA as a low single daily dose. Moreover, the potential risk to healthcare professionals related to the presence of IDA in patients’ fluids has so far not been tested.

The main research aim was to develop a rapid, sensitive, accurate and precise LC method with FL detection for the quantification of IDA in human plasma and urine, requiring small volume samples. For this purpose, the best chromatographic conditions for IDA quantification based on FL detection were selected. Next, various sample preparation protocols based on different extraction techniques, including LLE, SPE and SPME were evaluated. The developed LC-FL method was validated and successfully used for the calculation of IDA concentration profiles in both fluids after a single oral administration of the drug at a dose of 10 mg to a patient with RMA. Moreover, an evaluation of the potential risk of IDA exposure for healthcare workers and family members after the oral drug administration to the patient was undertaken and compared to an intravenous dosage of other anthracyclines.

## 2. Results and Discussion

As mentioned, IDA is commonly used in systemic cancer therapy for adult and pediatric patients [2,5,10]. This cytostatic drug can be administered both intravenously and orally in monotherapy, and as a component of various multi-drug therapies [2,6,9,11,13]. Depending on the dose and route of IDA administration, an appropriate analytical method for the specific pharmaceutical and/or clinical application is required. It should be noted that drug monitoring is recommended for IDA due to inter-individual variation, which makes difficult the adjustment of the proper dose to a specific patient. In effect, less effective anticancer therapy and increased side effects can be observed. These aspects should be seriously considered, especially for pediatric patients, because pharmaceutical and clinical data for this group are limited in the literature [12,13,26]. As mentioned in the Introduction, several methodologies have been reported in the literature for the quantification of IDA and its metabolites or other substances from the anthracyclines group in biological samples, and a more detailed description of LC methods was presented in Table S1. Most of them were developed more than 10 years ago when validation studies were less restrict. Additionally, many of these methods possess numerous disadvantages making them difficult to be used for many pharmaceutical and clinical applications. Thus, the aim of this work was to develop a reliable, sensitive and accurate procedure for the quantification of IDA in human plasma and urine samples as an interesting alternative to the methodologies described previously in the literature. Thus, multiple experiments were performed to develop a simple sample preparation procedure, and to establish the chromatographic conditions for IDA analysis by LC in biological fluids.

### 2.1. Optimization of the Sample Preparation

According to the literature data, most sample preparation procedures for the isolation of IDA from biological matrices were based on LLE using different mixtures of organic solvents like chloroform/heptanol [15], 2-propanol/chloroform [20], or dichloromethane (DCHM) [16] (Table S1). These protocols were applied following deproteinization [16] or an additional re-extraction into acidic solution was required [15]. In some cases, sample preparation was limited to the precipitation of proteins using acetonitrile (ACN) [19]. Moreover, some SPE protocols with C18 cartridges were described [6,7,9,14,17,18]. In the presented study, multiple IDA extraction procedures from human urine and plasma based on various techniques such as deproteinization, LLE, SPE and SPME were performed. During the experiments, the absolute recovery of IDA in both biological fluids was determined by comparing the peak responses of the extracted urine and plasma samples and non-extracted ones containing an analyte at the same concentrations of 10 and 100 ng/mL for urine and 5 and 25 ng/mL for plasma, respectively, each of which were measured in triplicate. Firstly, various extraction techniques for the isolation of IDA from urine were tested. Next, when the absolute extraction result for the compound of interest was above 50%, the same sample preparation protocol was used for plasma samples. In the case of all tested protein precipitation and LLE-based procedures, 2 mL of deproteinizing/eluting solvent was used. The sample was vortexed (30 s) and shaken mechanically for 10 min. After centrifugation for 7 min at 4500 rpm, the organic layer was transferred to another clean tube. A further sample preparation protocol was performed, as described in the Sample Preparation section. The obtained extraction results for IDA are summarized in Table 1. They indicate that deproteinization using ACN allowed an absolute recovery of 45.3 ± 4.6% to be obtained from urine samples. DCHM as the extracting agent was less effective (26.7 ± 2.2%), but after alkalization with 1 M NaOH, the recovery increased (37.2 ± 4.2%). LLE based on the mixture of chloroform and methanol (MeOH) (4:1, *v*/*v*) offered worse results (33.2 ± 3.9%). For this extracting solvent, alkalization decreased the efficiency (25.0 ± 4.9%). On the other hand, after using a mixture of chloroform and 1-propanol (4:1, *v*/*v*) as well as chloroform and 2-propanol (4:1, *v*/*v*), significantly higher absolute recoveries for IDA were calculated (71.1 ± 6.7% vs. 92.8 ± 9.9% for urine and 69.2 ± 6.3% vs. 89.1 ± 8.8% for plasma, respectively). For the mixture of 2-propranol and chloroform, it was observed that bringing the urine pH to alkaline by the addition of 1 M NaOH or phosphate buffer (pH 8.5) reduced the efficiency to 73.5 ± 8.6% and 72.7 ± 6.3% for urine and 75.1 ± 7.1% vs. 75.8 ± 8.2% for plasma, respectively.

Moreover, the use of ultrasonication (10 min, 37 °C) caused a further significant decrease in IDA extraction to 23.6 ± 2.9% for urine samples. This effect could be related to the loss of extracting solvents, especially chloroform. The above-presented data confirmed that LLE based on the mixture of chloroform:2-propanol (4:1, *v*/*v*) offered the most effective optimal extraction of IDA from both biological fluids.

Next, many SPE procedures to isolate IDA from both tested biological matrices were conducted using two types of cartridges—LiChrolut RP18 40 mg and hydrophilic-lipophilic balance (HLB) cartridge (30 mg). As it was mentioned, so far, only C18 cartridges were applied [6,7,9,14,17,18]. All SPE procedures were performed in the experimental conditions described in the Sample Preparation section, except for different sample modifications before SPE, and using other eluting solvents for IDA extraction. Therefore, before loading the urine or plasma samples into the SPE columns, the biological matrix was modified by the addition of 900 μL of redistilled water with 100 μL of phosphate buffer (pH 8.5) or 1 mL of 0.1 M HCl. Moreover, 950 μL of redistilled water with 50 μL of 1 M NaOH were added to the samples as the sample modifier. Firstly, for SPE with both C18 and HLB cartridges, water to purify the sample, and pure MeOH as an eluting agent were used. The obtained results confirmed that independent of the type of SPE cartridge used, the alkalization of the biological matrix before SPE negatively influences the IDA extraction process. For example, the absolute recovery results were 39.2 ± 1.3% and 46.3 ± 4.5% for C18 and HLB, respectively, when the phosphate buffer (pH 8.5) was added to the urine sample. After adding 0.1 M HCl as the pH sample modifier, significantly higher absolute recoveries for IDA were calculated (C18—94.6 ± 5.9% and 97.1 ± 5.5%; HLB—95.2 ± 3.7% and 99.0 ± 4.0%, for urine and plasma samples, respectively). This indicates that this stage of the procedure had an important influence on IDA extraction from both matrices independently of the type of SPE cartridges used. So far, the acidification of the sample matrix by 0.1 M HCl before SPE was not reported for IDA. Therefore, the sample modification described above was selected as the most optimal for further investigations. The results also confirmed that HLB columns provided slightly better results of IDA extraction than C18, and they were used in further research.

Next, the protocol based on the matrix modification with 0.1 M HCl was supported by ultrasonication before SPE with HLB (10 min, 37 °C). Nevertheless, it did not improve the efficiency for IDA when the values of absolute recoveries were more varied compared to those calculated without this process (95.0 ± 7.1% for urine and 98.5 ± 8.8% for plasma, respectively). Additionally, the efficiency of other eluting solvents was tested, such as a mixture of ACN:MeOH (1:1, *v*/*v*) and DCHM:2-propanol (1:1, *v*/*v*). However, the absolute recoveries for IDA were lower than those obtained with MeOH. Thus, pure MeOH was selected as the most effective eluting solvent for IDA from HLB cartridges.

During the optimization of the sample preparation procedure, the SPME extraction was also investigated. It is worth noting that there are no literature reports regarding the use of SPME as a technique for isolating IDA from human fluids. In the study, 96-well SPME brushes with C18 resin were used. All SPME procedures were conducted as described below: (1) the activation of C18 resin with 1 mL of MeOH:H_2_O (1:1; *v*/*v*) for 30 min, (2) washing with 1 mL of water for 10 s, (3) the modification of the sample matrix by the addition of 1 mL of 0.1 M HCl for urine and 0.5 mL for plasma before SPME, respectively, (4) the loading of the sample to the wells of a 96-well plate, (5) extraction for 100 min (shaking speed 850 rpm), (6) washing the brush fibers with deionized water for 10 s, (7) the desorption step with a desorbent for 100 min. A further sample preparation protocol was carried out under experimental conditions as described in the Sample Preparation section. In all SPME experiments, 0.1 M HCl and water were used as the eluting and washing agents, respectively. During the study, more attention was paid to the evaluation of the effect of various organic solvents used for the desorption of IDA from the C18 cartridges. The results confirmed that pure MeOH and the mixture of MeOH and ACN (1:1, *v*/*v*) resulted in inefficient IDA extraction (36.7 ± 1.9 and 37.9 ± 2.6% for urine samples, respectively). The values of the absolute recoveries of IDA were higher after using a mixture of ACN and water (1:1, *v*/*v*), chloroform:2-propranol and DCHM:2-propranol, each in the volume proportion (4:1, *v*/*v*). These desorbing agents gave comparable extraction results for IDA in the range of 51.6–52.2 and 52.2–55.3% for urine and plasma samples, respectively. The highest absolute recovery was achieved using a mixture of DCHM:2-propanol (1:1, *v*/*v*) (58.5 ± 2.7 and 59.2 ± 3.1% for urine and plasma samples, respectively). Additionally, better purification of the sample matrix was obtained for SPME compared to SPE, especially for urine samples.

The above-presented data indicate that SPME was less effective than SPE with C18 and HLB cartridges. The best SPME protocol was able to extract IDA with mid-range efficiency among the tested LLE procedures. On the other hand, 96 samples could be prepared simultaneously. In the case of the developed SPME procedure, this gives a mean preparation time of one sample about every 2 min. Moreover, the consumption of expensive organic solvents can be reduced if proper conditioning is provided. Taking into account the fact that the absolute recovery results for IDA in both biological fluids were stable, it might suggest that the developed SPME protocol can be used for specific pharmaceutical and clinical applications when a high dose of IDA is orally or intravenously administered to patients.

Summarizing the obtained results, during the optimization of the sample preparation for IDA by the LLE, SPE and SPME techniques, it should be noted that the highest efficiency was provided by SPE with HLB, which was carried out in acidic pH (0.1 M HCl) and using water and pure MeOH as the washing and eluting solvents, respectively. A detailed description of the developed SPE procedure was presented in the Sample Preparation section. The absolute recoveries for the I.S. were 98.3 ± 3.3% and 98.6 ± 3.5% for urine and plasma, respectively. It should also be emphasized that the amounts of urine (1 mL) and plasma (0.5 mL) were lower than in earlier published reports [6,7,9,14,18].

### 2.2. Optimization of LC Parameters

The optimization of chromatographic conditions for IDA separation by LC required the selection of an appropriate analytical column. According to the literature data, columns filled with an octadecyl phase dominated for the analysis of this substance [6,16,17,19,20], although octyl [14], phenyl [7] and cyan phases [9,15,18] were also applied. In the presented study, six stationary phases: two Discovery^®^ HS C18 of different lengths (150 and 250) (Supelco, Bellefonte, USA), Synergi 4μ Hydro-RP 80A 150 (Phenomenex, Torrance, CA, USA), Synergi 4μ MAX-RP 80A 150 (Phenomenex), InertSustain C18 150 (GL Sciences, Tokyo, Japan) and Hypersil BDS C18 (MZ-Analysentechnik, Mainz, Germany) were tested. Throughout the optimization process, all stationary phases were tested with the column temperature of 30 °C, the mobile phase flow rate of 1 mL/min, and the total analysis time of ≤10 min. Taking into account the signal intensity and symmetrical peak of IDA, the retention times of this compound and other anthracyclines considered as potential I.S.s, and the parameter of resolution, finally, as the most optimal for IDA determination in human urine and plasma, the Discovery^®^ HS C18 column (250 mm × 4.6 mm, 5 μm) was selected. Among the tested potential I.S.s, epirubicin, doxorubicin and DAU, each as hydrochloride salts, were analyzed. Finally, DAU was selected as the most optimal I.S. because of its signal response, symmetrical peak shape, and the appropriate retention time.

Next, the optimal composition of the mobile phase was investigated. In the study, mixtures of 0.1% aqueous formic acid (component A) and ACN (component B) in various volume proportions were tested (A—from 63 to 75%; B—from 37 to 25%). The best symmetry and peak shapes of the compounds of interest, the retention times and separate analytical signals for IDA and the I.S. from the peaks of the sample matrix were obtained when the chromatographic separation was carried out using the phase composition of A:B (67:33, *v*/*v*) for plasma samples and (68:32, *v*/*v*) for urine samples, respectively. Therefore, this phase composition was chosen as the optimal one for IDA separation.

Research on the conditions of IDA quantification was also carried out in the search for the optimal FL detection conditions. As it was mentioned, FL detectors are able to offer higher selectivity and sensitivity than UV. These parameters can be comparable to the values obtained by MS. Additionally, FL apparatuses, because of low cost and simple maintenance, are used in many laboratories. In the work, different wavelengths in the range of 460–491 nm for the excitation wave and from 540 to 555 nm for the emission wave were tested. The highest analytical signals for IDA were obtained with the simultaneous use of the excitation wavelength equal to 487 nm and the emission wavelength of 547 nm. Therefore, these parameters for IDA quantification were selected for further investigations.

Summarizing, the most optimal chromatographic parameters for IDA analysis in human urine and plasma samples were described in the Chromatographic Conditions section. The herein-developed LC-FL protocol allowed IDA concentrations to be detected and measured in both biological fluids in a shorter analysis run time (8 and 10 min for urine and plasma samples, respectively) than reported in previously published papers [14,15,16,17,18,19] (Table S1).

### 2.3. Validation of Analytical Method

The LC-FL method was completely validated in accordance with the Food and Drug Administration (FDA) and the International Conference of Harmonization of Technical Requirements for the Registration of Pharmaceuticals for Human Use (ICH) requirements [27,28] regarding linearity, selectivity, accuracy and precision, sensitivity, absolute recovery and stability. The obtained validation data have been presented in Materials and Methods (Section 3.6).

### 2.4. Application to Real Samples

To the best of our knowledge, in the scientific literature, there are limited numbers of papers reporting real plasma and urinary IDA profiles obtained from pediatric patients [12,13,26]. Thus, the probability of incorrect IDA administration should be considered, especially that individual differences additionally make it difficult to adjust the IDA dose to a specific patient.

**Figure 1:**
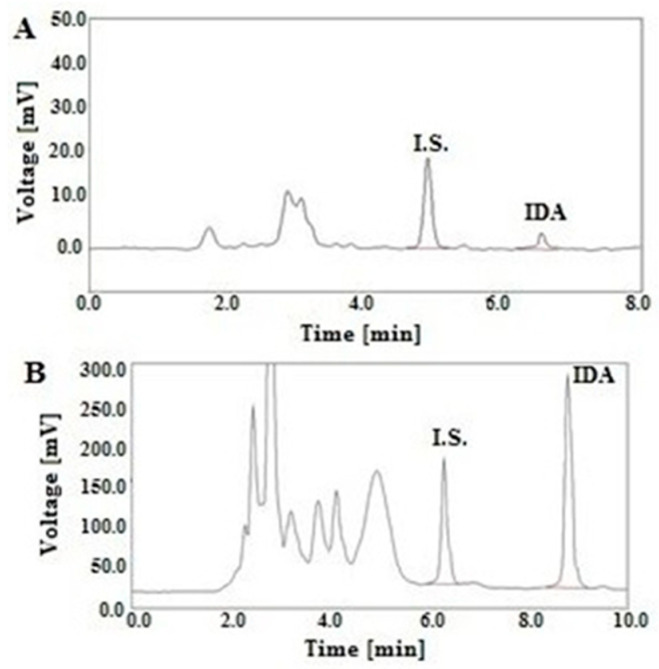
Representative LC chromatograms of a patient’s human plasma extract collected at 4 h (**A**), and a urinary sample collected between 12 and 20 h (**B**) after oral administration of IDA at a dose of 10 mg on the 1st day of the O-TIDA/O-TE therapy, according to the CWS-2006 protocol, at the following IDA levels: A—1.44 ng/mL of IDA and DAU (I.S.) at a concentration of 20 ng/mL; B—142.33 ng/mL of IDA and DAU (I.S.) at a concentration of 100 ng/mL.

In the study, the developed LC-FL method was used to measure the IDA levels in real human plasma and urine after oral drug administration of 10 mg to a child with RMA. The 17-year-old boy was treated according to the Cooperative Weichteilsarkom Studiengruppe (CWS)-2006 protocol, in which IDA was given at a dose of 10 mg once a day on days 1, 4, 7 and 10 in the scheme of TIDA/O-TE. The plasma and urine samples were collected on the 1st and the 10th day of the oncological treatment. The representative chromatograms of the plasma sample obtained at 4 h and the urinary sample collected at 12–20 h after the oral IDA administration are illustrated in Figure 1A,B, respectively.

**Figure 2:**
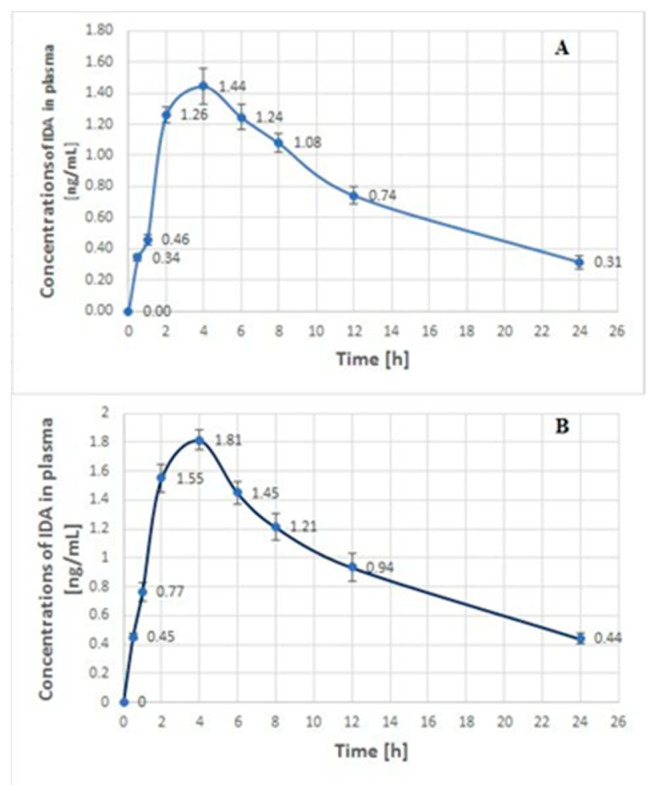
The concentration-time profiles of IDA in plasma samples (mean ± SD, *n* = 3) from a 17-year-old child with RMA after oral administration of IDA, each at a dose of 10 mg, on the 1st day (**A**) and the 10th day (**B**) of the O-TIDA/O-TE therapy, according to the CWS-2006 protocol.

**Figure 3:**
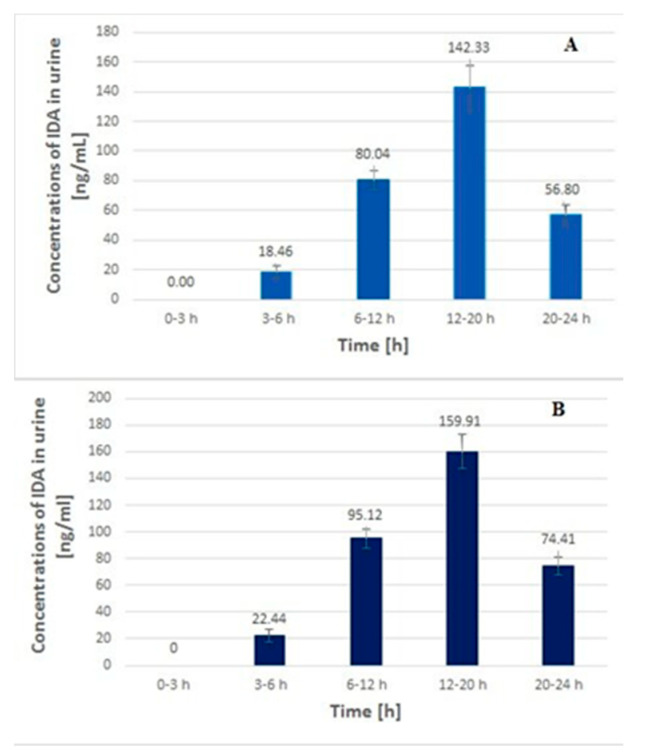
The concentration-time profiles of IDA in urine samples (mean ± SD, *n* = 3) from a 17-year-old child with RMA after oral administration of IDA, each at a dose of 10 mg, on the 1st day (**A**) and the 10th day (**B**) of the O-TIDA/O-TE therapy, according to the CWS-2006 protocol.

The calculated plasma and urinary concentration-time profiles of IDA in the samples obtained on the 1st day and the 10th day of the O-TIDA/O-TE treatment are shown in Figure 2 and Figure 3, respectively.

These data confirmed that the plasma concentration of IDA after both administrations increased to the maximum concentration (C_max_) of 1.44 ± 0.11 and 1.81 ± 0.10 ng/mL, respectively, at the time (T_max_) of 4 h. Subsequently, a slow decrease in the plasma analyte concentrations to 0.31 ± 0.03 and 0.44 ± 0.03 ng/mL, respectively, was observed at 24 h after drug administration. The calculated plasma concentrations of IDA and the parameters of T_max_ and C_max_ were consistent with the data previously reported in the literature [6,7]. This suggests that no significant interactions between IDA and other drugs (trofosfamide and etoposide) used in the O-TIDA/O-TE course were observed.

In the case of the urinary profiles of IDA, the analyte concentrations slowly increased to the highest levels of 142.33 ± 14.84 and 159.91± 4.78 ng/mL, obtained in the samples collected at 12–20 h. Next, these values decreased to the levels of 56.80 ± 6.8 and 74.41 ± 9.61 ng/mL, respectively, in the samples obtained at 20–24 h. These data indicate that the urinary IDA levels were significantly higher than those calculated in the plasma samples. For example, the concentrations in the urine sample obtained at 12–20 h were almost 100 and 90 times higher, respectively, than those calculated for the plasma samples collected at 4 h (Figure 2 and Figure 3, respectively). This may suggest that the potential risk of exposure for the hospital staff and the patient’s caregivers is significantly higher with regard to contact with the patient’s urine than with plasma if the appropriate rules of safety are not respected. This issue was earlier indicated by Sottani et al. [14], although no numerical data were reported. Real plasma and urinary profiles of doxorubicin after 4 h and 3 h intravenous administration at a dose of 40 and 20 mg/m^2^, respectively, were published by Maliszewska et al. [29]. Epirubicin profiles in a pediatric patient’s plasma and urine samples obtained after intravenous drug infusion at a dose of 150 mg/m^2^ were also reported by Treder et al. [30]. Those results indicated that the urinary concentrations of both cytostatics calculated in the samples collected at the end of the drug administration were significantly higher than those found at T_max_ in the plasma samples (more than 70 and 95× for doxorubicin and 32× for epirubicin, respectively). Taking into consideration the IDA profiles obtained in the present study, it can be concluded that the differences between the levels of plasma and urinary anthracycline are significant, independent of their chemical structures, dose and route of administration. Importantly, the probability of exposure for healthcare workers and family members significantly increases when anthracycline is administrated intravenously (urinary concentrations were detected at a level of µg/mL), but this factor should also be considered for high oral drug dosage. Therefore, protective tools should be used by hospital personnel and a patient’s caregivers, regardless of the form of anthracycline administered.

## 3. Materials and Methods

### 3.1. Reagents

Idarubicin hydrochloride (IDA) (>98% purity) was supplied by Cayman Chemical (Ann Arbor, MI, USA). Daunorubicin hydrochloride (DAU) (internal standard, I.S.) (>98% purity) was obtained from Tocris Bioscience (Bristol, UK). Acetonitrile (ACN) and methanol (MeOH) of HPLC grade were provided by J.T. Baker (Phillipsburg, NJ, USA). Hydrochloric acid (36%), sodium hydroxide (NaOH), disodium phosphate (Na_2_HPO_4_), sodium dihydrogen phosphate (NaH_2_PO_4_) and *ortho*-phosphoric acid (H_3_PO_4_) were supplied by POCH (Gliwice, Poland). Chloroform, 2-propanol and dichloromethane (DCHM) of analytical grade were obtained from Merck (Darmstadt, Germany). Formic acid was supplied by Sigma-Aldrich (St. Louis, MO, USA). For the SPE experiments, the J.T. Baker Spe-12G system (J.T. Baker, Greischeim, Germany) was applied. Select hydrophilic-lipophilic balance (HLB) SPE cartridges (30 mg, 1 mL) were from Supelco (Bellefonte, PA, USA), whereas LiChrolut RP18 SPE cartridges (40 mg, 1 mL) were from Merck. A manual 96-well SPME station and brushes with blades coated in C18 sorbent were supplied by Pas Technologies, (Magdala, Germany). The deionized water used in all experiments was obtained from Milli-Q equipment (Millipore, Bedford, MA, USA). The control plasma and urine samples were obtained from healthy volunteers.

### 3.2. Chromatographic Conditions

The chromatographic measurements of plasma and urine samples were performed on an ACME 9000 system (Younglin Instrument Corporation, Anyang, Republic of Korea). The system consisted of a SP 930D pump, autosampler, CTS30 thermostat module with AutoChro-3000 software and a RF- 551 fluorescence detector (Shimadzu, Kyoto, Japan) operating at the excitation and emission wavelengths set at 487 nm and 547 nm, respectively. The LC separation was achieved on a Discovery HS C18 column (Supelco, 250 × 4.6 mm, 5 µm) set at 30 °C. All analyses were carried out at a flow rate of 1 mL/min with a mobile phase containing ACN and 0.1% formic acid in water (33:67, *v*/*v*) for plasma and (32:68, *v*/*v*) for urine samples, respectively.

### 3.3. Standard Solutions

The stock standard solutions of IDA (500 µg/mL) and the I.S. (1 mg/mL) used were prepared separately. The exact amount of each compound (5 mg of IDA and 10 mg of I.S.) were weighed and dissolved in 10 mL of MeOH. Subsequently, the stock standard solutions of IDA were dissolved in MeOH to obtain working solutions with the final concentrations: 100, 10, 1 ng/mL. The working standard solution of the I.S. at a concentration of 1 µg/mL was prepared in the same way as above. All the standard solutions of IDA and the I.S. were kept in the dark at −20 °C.

### 3.4. Plasma and Urine Standards

For the calibration samples (CSs) and quality control samples (QCs) a volume of 1 mL of blank urine and a volume of 0.5 mL of blank plasma were used. To prepare the calibration samples for blank urine and plasma samples, appropriate amounts of working standard solutions of IDA and the I.S. were added. The IDA concentrations in the urinary CSs for the calculation of the calibration curve were 0.25, 0.5, 2, 5, 10, 25, 50, 100, 150 and 200 ng/mL, while the I.S. concentration was 100 ng/mL. The IDA levels in the plasma CSs for establishing the calibration curve were 0.1, 0.25, 0.5, 1, 2, 5, 7.5, 10, 15, 25 and 50 ng/mL, and the I.S. level was 20 ng/mL. The urinary and plasma QCs were prepared at the three levels of IDA: low (LQC), middle (MQC) and high (HQC). For urine, the LQC, MQC and HQC contained IDA at concentrations of 50, 100 and 150 ng/mL, respectively, and the I.S. level was 100 ng/mL. The plasma QCs were prepared at the levels of 0.5, 5 and 15 ng/mL for IDA and at a concentration of 20 ng/mL for the I.S., respectively.

### 3.5. Sample Preparation

To 1 mL of human urine and to 0.5 mL of human plasma, the IDA solution at a range of concentrations: 0.25–200 ng/mL for urine samples and 0.1–50 ng/mL for plasma samples, was added. Furthermore, the I.S. solution at a concentration of 100 ng/mL and 20 ng/mL, respectively for urine and plasma samples, was spiked. The next step was vortex-mixing for 30 s. Then 0.1 M HCl at a volume of 1 mL to urine samples and 0.5 mL to plasma samples was added. After being stirred again for 30 s, the samples were shaken mechanically for 10 min and at the next stage, were centrifuged for 7 min at 4500 rpm. Following the centrifugation, SPE was carried out based on HLB 30 mg cartridges previously activated with 2 mL of MeOH and 2 mL of redistilled water. Then, the urine and plasma samples were washed with 1 mL of redistilled water and dried in a vacuum for 5 min. The elution was carried out for the urine and plasma samples with 1 mL of MeOH into clean glass tubes. Following evaporation to dryness at 45 °C (centrivap concentrator ST-02202, Labconco, Kansas City, MO, USA), the residue was dissolved in 70 μL of ACN:water (1:1, *v*/*v*). After stirring for 30 s, the sample was transferred to an Eppendorf tube. The last stage was centrifuging for 7 min at 10,000 rpm and then the sample was transferred to the inserts and 20 µL analyzed by LC-FL.

### 3.6. Validation of Analytical Method

The developed LC-FL method for IDA determination in urine and plasma was fully validated in accordance with the FDA and ICH guidelines [27,28]. For the method validation purposes, the urinary and plasma calibration samples (CSs) and quality control samples (QCs) at three levels of IDA: low quality control (LQC), middle quality control (MQC) and high quality control (HQC), were prepared as described in Plasma and Urine Standards, and treated in the same manner as described in Sample Preparation and Chromatographic Conditions.

#### 3.6.1. Calibration Curve

For the calculation of the calibration curves in urine and plasma, six independent series of CSs containing IDA at eight concentration levels were spiked with the I.S. at concentrations of 100 ng/mL (urine) and 20 ng/mL (plasma). These CSs were processed and analyzed using the developed LC-FL method within one day. In both body fluids, the calibration curves were constructed using a least-squares linear regression model by plotting peak area ratios of IDA to the I.S. against the concentration of IDA. The obtained results for the urine and plasma samples are listed in Table 2.

#### 3.6.2. Linearity

To evaluate the linearity, blank urine and plasma samples were spiked with working standard solutions of IDA in order to obtain eight concentration levels over the range of 0.25–200 ng/mL and 0.1–50 ng/mL as well as the I.S. working solution to obtain the levels of 100 ng/mL and 20 ng/mL in urine and plasma, respectively. These CSs were analyzed on the same day. Concentrations were back-calculated from the corresponding calibration curves. As it is shown in Table 2, the linearity was confirmed by high values of the correlation coefficient (R^2^) of 0.9997 and 0.9998 in the considered urinary and plasma concentration ranges.

#### 3.6.3. Selectivity

**Figure 4:**
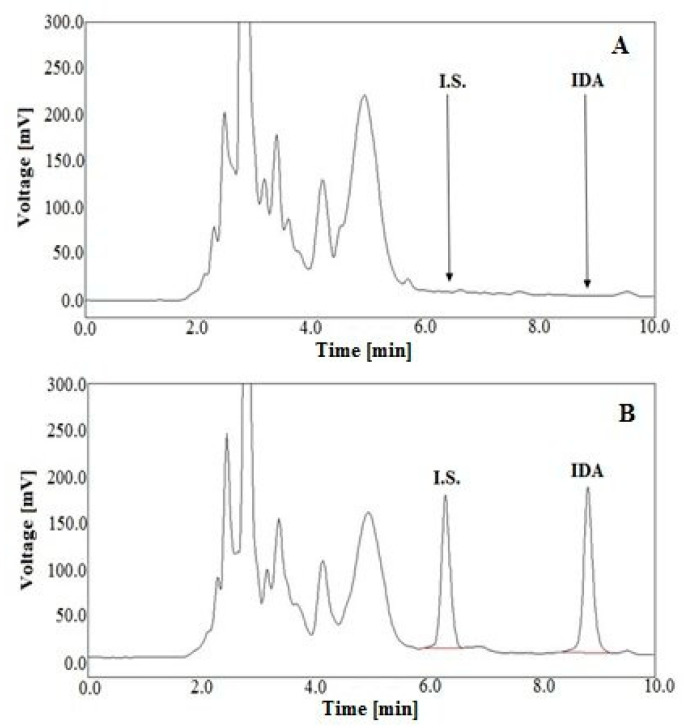
Representative LC chromatograms of blank human urine extracts (**A**), and a urine sample spiked with IDA at the level of 100 ng/mL and DAU (I.S.) at a concentration of 100 ng/mL (**B**), after SPE with HLB cartridges.

The selectivity of the developed method was verified by analyzing blank samples of urine and plasma, without being enriched with IDA and the I.S., and the samples which were spiked with these substances. The obtained chromatograms were compared for the absence of interference of the matrix with analytes (*n* = 6). Standard chromatograms corresponding to blank urine extracts and a urine extract sample enriched with IDA at a concentration of 100 ng/mL and the I.S. at the level of 100 ng/mL are shown in Figure 4A,B, respectively.

**Figure 5:**
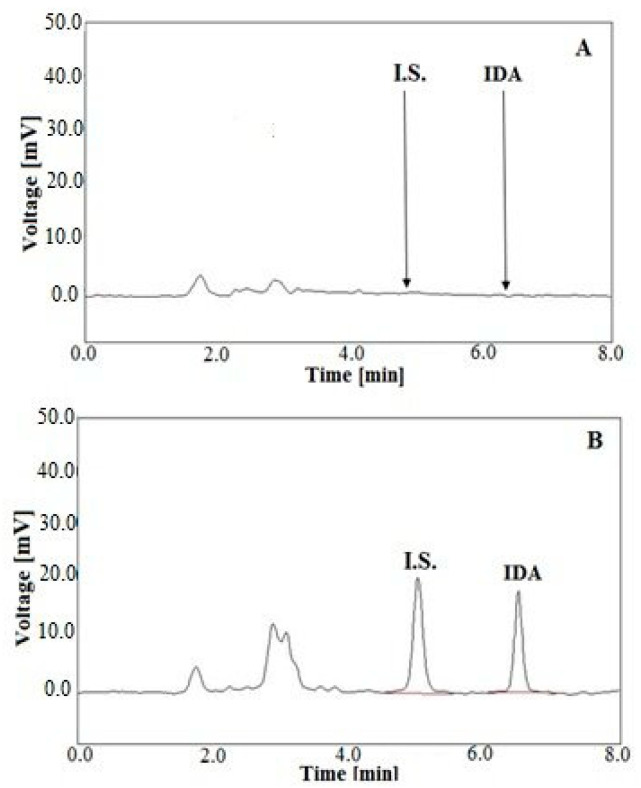
Representative LC chromatograms of blank human plasma extracts (**A**), a plasma sample spiked with IDA (15 ng/mL) and DAU (I.S.) at a concentration of 20 ng/mL (**B**), after SPE with HLB cartridges.

Representative chromatograms of blank plasma extracts and a plasma extract sample spiked with IDA at a concentration of 15 ng/mL and the I.S. at the level of 20 ng/mL are shown in Figure 5A,B, respectively.

Comparing these chromatograms, it was found that there are no interferences between the peaks from the urine and plasma matrices and IDA and the I.S., which means it can be stated that the developed LC-FD method is selective.

#### 3.6.4. Limit of Detection and Limit of Quantification

The LOD was estimated as the concentration of the sample at which the ratio of the values of the analyte signal to the baseline noise level (S/N) were 3:1. In the study, the LOD values in urine and plasma were found to be 0.125 ng/mL and 0.05 ng/mL, respectively (*n* = 6) (Table 2). The lower limit of quantification (LLOQ) represented the smallest concentration for which the signal from the detector was 10 times higher than the background noise, the precision was below 15% and the accuracy was in the range of 80–120%. In both biological fluids, the LLOQ parameter was calculated from six separately made replications, which were processed and measured within one day (intra-day assay) and between days within two months (inter-day assay) (Table 3). The data confirmed that the lowest concentrations in the calibration plots, regarded as the LLOQ, were at the levels of 0.25 ng/mL and 0.1 ng/mL in urine and plasma, respectively. These parameters were lower than the values of LOD and LOQ calculated for previously reported methods for IDA determination [6,9,14,15,16,17,18,19,20] (Table S1).

#### 3.6.5. Accuracy and Precision

The precision and accuracy of the method were estimated by performing an analysis with three replicates of LQC, MQC and HQC samples containing IDA at concentrations of 50, 100 and 150 ng/mL in urine and 0.5, 5 and 15 ng/mL in plasma, respectively, and the I.S. at the level of 100 and 20 ng/mL in the above matrices, respectively. These QCs were processed and measured on the same day (intra-day assay) and on six different days within 2 months (inter-day assay) (*n* = 6). Precision was expressed as the relative standard deviation (RSD (%)), whereas accuracy was determined as the percentage of difference between the found mean concentration and the nominal concentration. The intra- and inter-day precision and accuracy data are given in Table 3.

The intra-day validation results indicated that the values of RSD did not exceed 4.32 and 6.36% while the accuracy was in the range of 97.6–101.5% and from 96.4 to 99.2% for the urinary and plasma QCs, respectively. The inter-day precisions were between 0.15 and 9.50%, whereas the inter-day accuracies were from 99.1 to 106.4%. The presented data show that in both biological fluids, IDA met the generally accepted criteria for bioanalytical method validation at all QC concentration levels.

#### 3.6.6. Stability Study

The stability assessment of IDA in plasma and urine samples was performed under various conditions by the analysis of three replications of each QC at high, medium and low concentrations using the proposed LC methods. Thus, the short-term stability (at room temperature for 4 h), long-term stability (at −80 °C for two months), freeze/thaw stability (−80 °C to room temperature in a triple cycle), post-preparative stability (at 4 °C for 24 h) and autosampler stability (6 ± 2 °C, 24 h) of the analyte was evaluated. The assay was based on back-calculated levels and a comparison of the results with those obtained from freshly prepared samples. The sample was considered to be stable if the back-calculated concentration of the analyte was set within 100 ± 15% of the initial concentration. The obtained results, shown in Table S2, confirm that IDA and the I.S. were stable in plasma and urine samples under all storage conditions. These data are consistent with data previously reported in the literature [16,31].

### 3.7. Method Application

The above LC method was used to measure the levels of IDA in human plasma and urine after oral administration of the drug to a child with RMA. The patient was treated in the Department of Pediatrics, Hematology and Oncology at the University Clinical Center in Gdańsk. The Ethics Committee of the Medical University of Gdańsk (Gdańsk, Poland) gave the necessary approval for the test. Before starting the experiment, both parents and the patient read the protocol and gave formal written consent for participation in the study.

The patient was a 17-year-old boy (weighing 74.8 kg) with RMA of the head and neck area (stage III, very high risk group, VHRG) who was qualified to Arm B with O-TIDA/O-TE supportive maintenance chemotherapy according to the CWS-2006 protocol. The patient received IDA orally at a dose of 10 mg once a day on days 1, 4, 7 and 10. The samples of blood and urine were acquired twice at an interval of 10 days (the 1st and 10th day of the maintenance oncological therapy). About 5 mL of blood samples were collected into K_2_EDTA tubes in the following time compartments. The first sample was taken immediately before the drug administration at time 0. Subsequent plasma samples were collected at 0.5, 1, 2, 4, 6, 8, 12, 24 and 48 h after drug administration. About 10 mL of urine sample was collected at time 0 and at the time intervals: 0–3 h, 3–6 h, 6–12 h, 12–20 h, 20–24 h. The acquired blood and urine samples were kept frozen at −80 °C until the LC-FL analysis.

## 4. Conclusions

In the study, a simple, fast and sensitive LC-FL method in isocratic mode for the reliable quantification of IDA in plasma and urine samples was developed. Moreover, a new sample preparation protocol based on SPE with HLB cartridges was tested and compared to other LLE as well as SPE and SPME procedures. The developed SPE approach offered higher IDA extraction from both biological fluids and required a low sample volume (0.5 mL for plasma and 1 mL for urine). Moreover, the SPME procedure was tested for IDA for the first time. The developed SPE-LC-FL method was successfully applied for drug quantifications in human plasma and urine after oral IDA administration at a low dose to a pediatric patient. The proposed LC-FL method can be considered as an interesting alternative tool in pharmacokinetic and clinical investigations, in drug monitoring therapy as well as other techniques involving sensitive and reliable IDA quantitation. Additionally, attention was drawn to the requirement of complying with safety rules by healthcare workers and caregivers of patients in contact with the urine of a patient who has been administered IDA, regardless of the route of administration.

## Figures and Tables

**Table 1:** Absolute recovery results for IDA from urine and plasma after using various sample preparation procedures (*n* = 3) (mean ± SD).

**Deproteinizing Method/Liquid-Liquid Extraction**					**The Absolute Recovery of IDA (%)**	
					**Urine**	**Plasma**
ACN					45.3 ± 4.6	-
DCHM					26.7 ± 2.2	-
DCHM + 50 µL of 1 M NaOH					37.2 ± 4.2	-
Chloroform: MeOH (4:1, *v*/*v*)					33.2 ± 3.9	-
Chloroform: MeOH (4:1, *v*/*v*) + 50 µL of 1 M NaOH					25.0 ± 4.9	-
Chloroform: 1-propranol (4:1, *v*/*v*)					71.1 ± 6.7	69. 2 ± 6.3
Chloroform: 2-propranol (4:1, *v*/*v*)					92.8 ± 9.9	89.1 ± 8.8
Chloroform: 2-propranol (4:1, *v*/*v*) + 50 µL of 1 M NaOH					73.5 ± 8.6	75.1 ± 7.1
Chloroform: 2-propranol (4:1, *v*/*v*) + 100 µL of phosphate buffer (pH 8.5)					72.7 ± 6.3	75.8 ± 6.2
Chloroform: 2-propranol (4:1, *v*/*v*) + 100 µL of phosphate buffer (pH 8.5) + ultrasonication					23.6 ± 2.9	-
**Solid Phase Extraction (SPE)**						
**SPE Cartridge**	**Sample Matrix Modification**		**Washing Agent**	**Eluting Solvent**	**The Absolute Recovery of IDA (%)**	
					**Urine**	**Plasma**
C18 40 mg	900 µL of H_2_O + 100 µL of phosphate buffer (pH 8.5)		Water	MeOH	39.2 ± 1.3	-
	0.1 M HCl				94.6 ± 5.9	97.1 ± 5.5
HLB 30 mg	900 µL of H_2_O + 100 µL of phosphate buffer (pH 8.5)		Water	MeOH	46.3 ± 4.5	-
	950 µL of H_2_O + 50 µL of 1 M NaOH				56.2 ± 4.8	52.2 ± 4.9
	0.1 M HCl				95.2 ± 3.7	99.4 ± 4.0
	0.1 M HCl + ultrasonication				95.0 ± 7.1	98.5 ± 8.8
	0.1 M HCl			ACN:MeOH (1:1, *v*/*v*)	89.2 ± 5.9	94.6 ± 6.1
				DCHM:2-propranol (1:1, *v*/*v*)	92.1 ± 3.8	95.9 ± 4.9
**Solid Phase Microextraction (SPME)**						
**SPME Cartridge**	**Eluting Solvent**	**Washing Agent**	**Desorbing Solvent**		**The Absolute Recovery of IDA (%)**	
					**Urine**	**Plasma**
C18	0.1 M HCl	Water	MeOH		36.7 ± 1.9	-
			ACN: MeOH (1:1, *v*/*v*)		37.9 ± 2.6	-
			ACN: H_2_O (1:1, *v*/*v*)		51.6 ± 4.7	52. 2 ± 5.3
			Chloroform: 2-propranol (4:1, *v*/*v*)		52.2 ± 5.7	54.8 ± 5.5
			DCHM: 2-propranol (4:1, *v*/*v*)		52.0 ± 2.9	55.3 ± 3.4
			DCHM: 2-propranol (1:1, *v*/*v*)		58.5± 2.7	59.2 ± 3.1

HLB—hydrophilic-lipophilic balance; ACN—acetonitrile; MeOH—methanol; DCHM—dichloromethane.

**Table 2:** Summary of the validation data for IDA quantification in human plasma and urine by the SPE-LC-FL method (*n* = 6).

	Urine	Plasma
Linearity	0.25–200 ng/mL	0.1–50 ng/mL
Equation parameter		
Slope	0.0109 ± 0.00007	0.061 ± 0.0003
Intercept	0.0025 ± 0.0068	0.0041 ± 0.0069
Standard error	0.0139	0.0150
Correlation coefficient (R^2^)	0.9997	0.9998
LOD (ng/mL)	0.125	0.05

**Table 3:** Intra-day and inter-day precision and accuracy for the determination of IDA in plasma and urine samples by the SPE-LC-FD method (*n* = 6), respectively.

Urine					Plasma			
Intra-Day (*n* = 6)								
	Concentration (ng/mL)		Precisio RSD (%)	Accuracy (%)	Concentration (ng/mL)		Precision RSD (%)	Accuracy (%)
	Spiked (ng/mL)	Found (Mean ± SD)			Spiked (ng/mL)	Found (Mean ± SD)		
LLQC	0.25	0.26 ± 0.02	8.82	103.4	0.1	0.11 ± 0.01	9.09	110.0
LQC	50	49.16 ± 2.13	4.32	99.3	0.5	0.48 ± 0.03	6.38	96.4
MQC	100	97.63 ± 2.88	2.95	97.6	5	4.96 ± 0.20	4.00	99.2
HQC	150	152.22 ± 2.28	1.50	101.5	15	14.58 ± 0.42	2.90	97.2
**Inter-day (*n* = 6)**								
LLQC	0.25	0.26 ± 0.03	9.78	105.2	0.1	0.11 ± 0.01	9.09	110.0
LQC	50	49.79 ± 2.02	4.06	99.6	0.5	0.53 ± 0.05	9.50	106.4
MQC	100	100.42 ± 4.04	4.02	100.4	5	4.95 ± 0.07	1.48	99.1
HQC	150	149.79 ± 2.02	1.35	99.9	15	15.02 ± 0.02	0.15	100.1

## Supplementary Materials

The following are available online, Figure S1: Chemical structure of IDA (A) and DAU (I.S.) (B), Table S1: Comparison of LC methods for the quantification of IDA in biological samples reported in the literature, Table S2: Results of a stablity study for IDA in human plasma and urine under various experimental conditions.
