# Supplementary material for: Sensitive Analysis of Idarubicin in Human Urine and Plasma by Liquid Chromatography with Fluorescence Detection: An Application in Drug Monitoring
Source: Molecules. 2020 Dec 9;25(24):5799. doi: 10.3390/molecules25245799 (PMC7764277; doi:10.3390/molecules25245799)
Supplement: Supplementary file 1 [file molecules-25-05799-s001.pdf]

molecules

MDPI

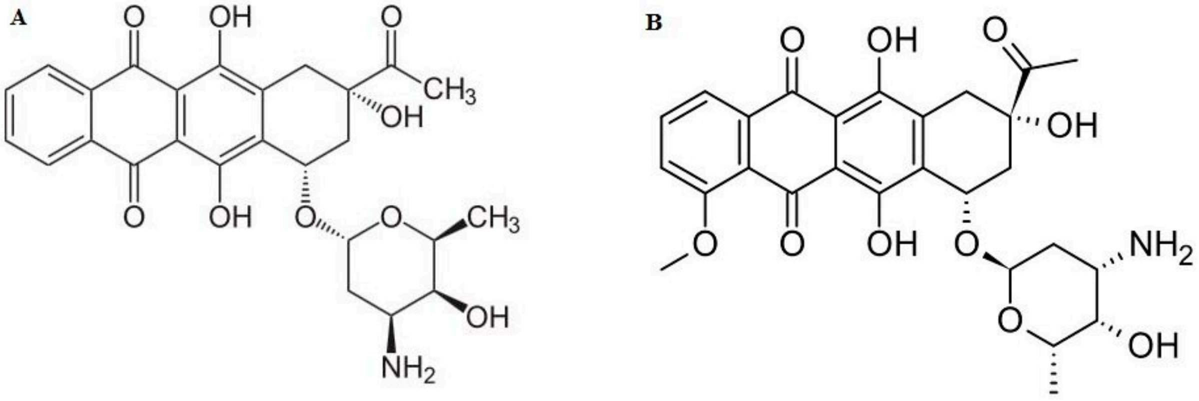

Figure S1: Chemical structure of IDA (A) and DAU (I.S.)

molecules MDPI

Table S1. Comparison of LC methods for the quantification of IDA in biological samples reported in the literature

Biological matrix	Determined substances	Extraction procedure	Absolute recovery (%)	Chromatographic method				Range of the linearity	LOD (ng/mL)	LOQ (ng/mL)	Ref.
				Stationary phase	Mobile phase	Detection conditions	Total analysis time (min)				
Human plasma 2 mL	IDA and idarubicinol	SPE C18 *Eluting solvent*: 3 NH,PO_4:ACN: H_2O:$(C_2H_5)N$ (20:40:39.95:0.05, *v/v/v/v*)	No data	C18 (250 x 4.6 mm, 5 μm)	*Isocratic elution:* 3 M H_3PO_4:ACN: H_2O: TEA (20:40:39.95:0.05, *v/v/v/v*)	FL λ_{ex} = 250 nm λ_{em} = 570 nm	No data	0.1-50 ng/mL	0.1 ng/mL	No data	[6]
Human plasma 1 mL and urine 50-100 μL	IDA and idarubicinol	Deproteinization with MeOH for plasma; dilution with water for urine before SPE with Isolute C18 *Eluting solvent*: 0.25 M HCl in MeOH	No data	Waters μBondapak phenyl column (300 x 3.9 mm, no data)	*Isocratic elution:* 0.4 M Ammonium formate (pH 4.0) : ACN (7:3, *v/v*)	FL λ_{ex} = 254 nm λ_{em} = 550 nm	No data	1-100 ng/mL for plasma No data for urine	No data	No data	[7]
Human plasma	IDA and idarubicinol	SPE with C18 *Eluting solvent*: No data	84	Supelcosil LC-CN (250 x 4.6 mm, 5 μm)	*Isocratic elution:* 10 mM KH_2PO_4 : ACN (pH 2.5) no data	FL λ_{ex} = 470 nm λ_{em} = 580 nm	No data	1.05-350 ng/mL	0.1 ng/mL	0.3 ng/mL	[9]

1-2 mL; Cerebrospinal fluid 1-2 mL											
Human urine 5 mL	IDA DAU Doxorubicin Epirubicin	SPE with Bond Elut C18 *Eluting solvent*: DCHM:2-propanol (1:1, *v/v*) pH 7	90.7	BDS C8 (150 x 4.6 mm, 5 µm)	*Gradient elution:* Component A: 0.1% HCOOH in H_2O:ACN:MeOH (70:25:5, *v/v/v*), Component B: 0.1% HCOOH in H_2O:ACN (70:30, *v/v*)	LC/MS/MS $m/z = 498$ $m/z = 291$	25	100-2000 ng/mL	10 ng/mL	30 ng/mL	[14]
Human plasma 0.5 mL	IDA DAU Doxorubicin Epirubicin and their 13-dihydrometab olites	LLE with chloroform: 1-heptanol (9:1, *v/v*) at pH 8.4 and re-extraction into 0.1 M H_3PO_4	93-109	Supelcosil LC-CN (250 x 4.6 mm, 5 µm)	*Isocratic elution:* 50 mM NaH_2PO_4: ACN (65:35, *v/v*) adjusted to pH 4.0 with H_3PO_4	FL $\lambda_{ex} = 230$, 254 and 480 nm $\lambda_{em} = 560$ nm	15	0.4-10000 ng/mL	0.4 ng/mL	0.4 ng/mL	[15]
Human plasma 400 µL and saliva 200 µL	IDA DAU Doxorubicin Epirubicin	Deproteinization with ethanol, followed by LLE extraction with DCHM at pH 8.5 (phosphate buffer)	81.7-87.4 for plasma, 76.0-77.4 for saliva	Purospher Star RP-18 (150 x 4.6 mm, 5 µm)	*Gradient elution:* Component A: 0.1% formic acid in water	FL $\lambda_{ex} = 480$ nm $\lambda_{em} = 555$ nm	14	1-1000 ng/mL for both fluids	0.3 ng/mL for both fluids	1 ng/mL for both fluids	[16]

	and their 13-dihydrometabolites				Component B: 0.1% formic acid in ACN						
Human serum 0.5 mL	IDA DAU Doxorubicin Epirubicin and three their 13-dihydro-metabolites	SPE with Bond Elut C18 *Eluting solvent*: Chloroform: 2-propanol (4:1 *v*/*v*)	85-105	Symmetry C18 (150 x 1 mm, 3.5 µm)	*Isocratic elution:* 5 mM ammonium formate buffer (pH 3.0) :ACN (70:30, *v*/*v*)	ESI-MS $m/z = 291$ $m/z = 333$	30	5-2000 ng/mL	1 ng/mL	5 ng/mL	[17]
Human plasma 0.5-2 mL Urine no data	IDA Idarubicinol 4-Demotoxy-daunomycinone	SPE with Bondelut C18 *Eluting solvent*: H_2O:MeOH (3:1, *v*/*v*) and 0.03 M H_3PO_4 in MeOH	91.5	Cyanopropyl column (250 x 4.6 mm, 5 µm)	*Gradient elution:* Component A: 10 mM KH_2PO_4: ACN (78:22, *v*/*v*) Component B: [10 mM KH_2PO_4 + 0.006 M H_3PO_4]: ACN (30:70, *v*/*v*)	FL $\lambda_{ex} = 470$ nm $\lambda_{em} = 580$ nm	20	0.14-35.3 ng	0.2 ng/mL	No data	[18]
Rat plasma 100 µL	IDA and idarubicinol	Deproteinization with ACN	95.6	LiChrospher 100 RP-18 (250 x 4 mm, 5 µm)	*Isocratic elution:* H_2O:ACN:THF: H_3PO_4:TEA (312:165:20:1:2, *v*/*v*/*v*/*v*/*v*) adjusted to pH 2.2 with 5 M HCl	FL $\lambda_{ex} = 485$ nm $\lambda_{em} = 542$ nm	15	1-500 ng/mL	0.25 ng/mL	No data	[19]

Rabbit plasma 0.5 mL Heart, liver and kidney tissues: 100-500 mg	IDA and idarubicinol	LLE chloroform: 2-propanol (9:1, *v*/*v*) at pH 8.2 (0.5 M Na_2HPO_4)	92.2	Ultracarb 5 ODS (150 x 4.6 mm, 5μm)	*Isocratic elution:* H_2O:ACN:THF: H_3PO_4:TEA (68.4:28:3:0.4:0.2, *v*/*v*/*v*/*v*/*v*) adjusted to pH 2.2 with 5 M HCl	FL λ_{ex} = 485 nm λ_{em} = 560 nm	No data	2-500 ng/mL	No data	2 ng/mL	[20]
**Human plasma 0.5 mL Urine 1 mL**	**IDA**	**pH modification with 0.1 HCl before SPE with Supel Select HLB** ***Eluting solvent:*** **MeOH**	**99.4 for plasma 95.2 for urine**	**Discovery HS C18 (150 × 4.6 mm, 5 μm)**	***Isocratioc elution*** **ACN:0.1% formic acid in water: (33:67, *v*/*v*) for plasma (32:68, *v*/*v*) for urine**	**FL λ_{ex} = 487 nm λ_{em} = 547 nm**	**8 for plasma 10 for urine**	**0.1-50 ng/mL for plasma 0.25-200 ng/mL for urine**	**0.05 ng/mL for plasma 0.125 ng/mL for urine**	**0.1 ng/mL for plasma 0.25 ng/mL for urine**	**Method in this study**

ACN – acetonitrile; DAU – daunorubicin; IDA – idarubicin; LLE – Liquid-liquid extraction; MeOH – methanol; SPE – solid phase extraction; TAE – trimethylamine; THF – tetrahydrofuran

molecules

MDPI

Table S2. Results of a stablity study for IDA in human plasma and urine under various experimental conditions

Storage conditions	QC	Conc. added (ng/mL)	Conc. found * (ng/mL)	Precision RSD (%)	Accuracy (%)
			Urine		
Long-term stability (-80°C, 2 months) stability	LQC	50	47.3 ± 4.8	10.1	94.6
	MQC	100	102.3 ± 8.4	8.2	102.4
	HQC	150	148.7 ± 10.3	6.9	99.1
Three freeze-thaw cycles stability	LQC	50	46.7 ± 5.4	11.6	93.4
	MQC	100	101.6 ± 8.2	8.1	101.6
	HQC	150	152.1 ± 10.5	6.9	101.4
Ambient storage (25°C, 12 h)	LQC	50	51.3 ± 4.1	8.0	102.6
	MQC	100	98.9 ± 6.6	6.7	98.9
	HQC	150	148.9 ± 8.4	5.6	99.3
Post-preparative storage (4°C, 24 h)	LQC	50	48.3 ± 4.6	9.5	100.6
	MQC	100	103.5 ± 7.2	6.9	103.5
	HQC	150	151.7 ± 7.9	5.2	101.1
Auto sample stability (6 ± 2°C, 24 h)	LQC	50	50.3 ± 3.9	7.7	99.6
	MQC	100	97.9 ± 6.8	6.9	97.9
	HQC	150	149.5 ± 8.2	5.5	99.7
			Plasma		
Long-term stability (-80°C, 2 months) stability	LQC	0.5	0.49 ± 0.05	10.2	98.0
	MQC	5	4.44 ± 0.33	7.4	88.8
	HQC	15	15.82 ± 1.01	6.4	105.5
Three freeze-thaw cycles stability	LQC	0.5	0.53 ± 0.04	7.5	106.0
	MQC	5	5.15 ± 0.26	5.0	103.0
	HQC	15	14.71 ± 0.78	5.3	98.1
Ambient storage (25°C, 12 h)	LQC	0.5	0.51 ± 0.03	5.9	102.0
	MQC	5	4.89 ± 0.25	5.1	97.8
	HQC	15	14.22 ± 0.68	4.8	94.8
Post-preparative storage (4°C, 24 h)	LQC	0.5	0.47 ± 0.04	8.5	94.0
	MQC	5	5.34 ± 0.37	6.9	106.8
	HQC	15	15.66 ± 0.77	4.9	104.0
Auto sample stability (6 ± 2°C, 24 h)	LQC	0.5	0.52 ± 0.03	5.8	104.0
	MQC	5	5.07 ± 0.13	2.6	101.4
	HQC	15	14.37 ± 0.57	4.0	95.8

* mean ± SD value from three samples
